# Effect of Human Adenovirus 36 on Response to Metformin Monotherapy in Obese Mexican Patients with Type 2 Diabetes: A Prospective Cohort Study

**DOI:** 10.3390/v15071514

**Published:** 2023-07-07

**Authors:** José Carlos Tapia-Rivera, Héctor Eduardo Mendoza-Jaramillo, Christian Octavio González-Villaseñor, Mario Ramirez-Flores, José Alonso Aguilar-Velazquez, Andres López-Quintero, Edsaúl Emilio Pérez-Guerrero, María de los Ángeles Vargas-Rodriguez, Itzae Adonai Gutiérrez-Hurtado, Erika Martínez-López

**Affiliations:** 1 Departamento de Ciencias Básicas para la Salud, Centro Universitario del Sur, Universidad de Guadalajara, Ciudad Guzmán 49000, Mexico; jose.tapia@cusur.udg.mx (J.C.T.-R.); mendozahctor@gmail.com (H.E.M.-J.); christiang@cusur.udg.mx (C.O.G.-V.); 2 Centro de Investigaciones Biomédicas, Universidad de Colima, Colima 28040, Mexico; mario_ramirez@ucol.mx; 3 Departamento de Morfología, Centro Universitario de Ciencias de la Salud, Universidad de Guadalajara, Guadalajara 44340, Mexico; josealonso.aguilarvelazquez@academicos.udg.mx; 4 Departamento de Biología Molecular y Genómica, Centro Universitario de Ciencias de la Salud, Universidad de Guadalajara, Guadalajara 44340, Mexico; andres.lopezq@academicos.udg.mx (A.L.-Q.); edsaul.perezg@academicos.udg.mx (E.E.P.-G.); 5 Doctorado en Ciencias de la Nutrición Traslacional, Centro Universitario de Ciencias de la Salud, Universidad de Guadalajara, Guadalajara 44340, Mexico; angeles.vargas8399@alumnos.udg.mx; 6 Instituto de Nutrigenética y Nutrigenómica Traslacional, Centro Universitario de Ciencias de la Salud, Universidad de Guadalajara, Guadalajara 44340, Mexico

**Keywords:** human adenovirus-36, type 2 diabetes, response to metformin

## Abstract

Human adenovirus 36 (HAdV-36) has been associated with obesity and changes in glucose and lipid metabolism. The virus has been reported to increase insulin sensitivity and paradoxically promote weight gain. Because of its effects on metabolism, infection with the virus could alter the response to several drugs used to treat type 2 diabetes (DM2), such as metformin. The aim of this study was to test whether HAdV-36 affects the response to metformin in a group of obese patients with DM2. Methods: In a prospective cohort study, 103 obese patients with newly diagnosed DM2 were divided into two groups based on their HAdV-36 seropositivity (+HAdV-36 and −HAdV-36). Weight, glucose, cholesterol, triglycerides, body mass index, body fat percentage, and waist and hip circumference were measured and compared in both groups at baseline and after 45 days of metformin treatment. Results: Only glucose was significantly lower in the +HAdV-36 group at baseline, while all other variables were similar between the two study groups. After 45 days of follow-up, it was observed that the effect of metformin did not differ between the groups, but the variables improved significantly after treatment. Conclusions: In this study, we did not find that HAdV-36 had an effect on the response to metformin in obese patients with DM2.

## 1. Introduction

Type 2 diabetes (DM2) is a chronic disease that has reached epidemic levels. Despite major efforts to control the disease, the number of cases is steadily increasing, with the number of patients with DM2 estimated to be 537 million worldwide in 2021 and projected to rise to 783 million by 2045 [1]. Therefore, much effort has been put into the prevention and control of DM2 due to the magnitude of the disease.

The risk of morbidity and mortality associated with DM2 is reduced by early diagnosis and timely initiation of treatment [2]. The appropriate management of DM2 requires a multifaceted approach that includes a healthy lifestyle with good dietary habits and regular physical activity, as well as a therapeutic regimen tailored to the needs and characteristics of the patient, taking into account comorbidities and treatment goals [3].

Among the most widely used drugs in the management of DM2, metformin has been considered first-line pharmacotherapy for more than two decades [4,5]. In addition to being effective, safe and inexpensive, metformin has beneficial effects on glycated hemoglobin (A1C), weight, lipids and insulinemia and reduces the risk of cardiovascular mortality [3,4,6]. While there is a large body of evidence supporting the benefits of this drug in controlling DM2, it is estimated that approximately 35% of patients do not achieve glycemic control on metformin monotherapy [7,8].

Pharmacogenetics have dominated studies aimed at explaining variation in patient response to metformin. For example, variants in the genes *SLC2A2*, *SLC22A1*, *SLC47A1*, *SLC47A2* and others have been associated with changes in drug response. [7,8,9]. However, other factors may be associated with metformin response, such as human adenovirus-36 (HAdV-36), which increases insulin sensitivity in cell and animal models and may, therefore, influence metformin response in patients with DM2 [10].

HAdV-36 was isolated for the first time in 1978 from a human fecal sample; however, the peak of research on this virus occurred in early 2000, when it was discovered that HAdV-36 was associated with obesity in humans but paradoxically also lowered cholesterol and triglyceride levels [11,12]. These early findings sparked interest in studying the relationship between HAdV-36 and metabolism. It was soon discovered that HAdV-36, while promoting weight gain, also improved insulin sensitivity, in the same way as thiazolidinediones (TZDs), which, while improving insulin sensitivity and lipid profile, also promote weight gain and subcutaneous adipose tissue. This coincidence in the effects between HAdV-36 and TZDs gave the guideline to consider that both generated similar metabolic changes [13,14].

It was soon discovered that, like TZDs, HAdV-36 upregulates peroxisome proliferator-activated receptors γ (PPARγ), which increases levels of adiponectin and the glucose transporters GLUT1 and GLUT4, thereby improving insulin sensitivity. However, PPARγ activation also favors adipogenesis, adipocyte differentiation and proliferation, as well as fatty acid absorption and storage, which explains the improvement in lipid profile and weight gain [14,15,16].

The relationship between TZD and metformin has been extensively studied and there is evidence that the combination of pioglitazone with metformin results in better-glycosylated hemoglobin (HbA1C), decreased markers of inflammation and lower cholesterol and triglyceride levels, conferring greater cardiovascular protection than metformin as monotherapy [17,18,19]. HAdV-36, by regulating metabolic pathways similar to TZDs, may influence the response to metformin in patients with DM2. However, to our knowledge, no study has investigated this relationship.

**Figure 1:**
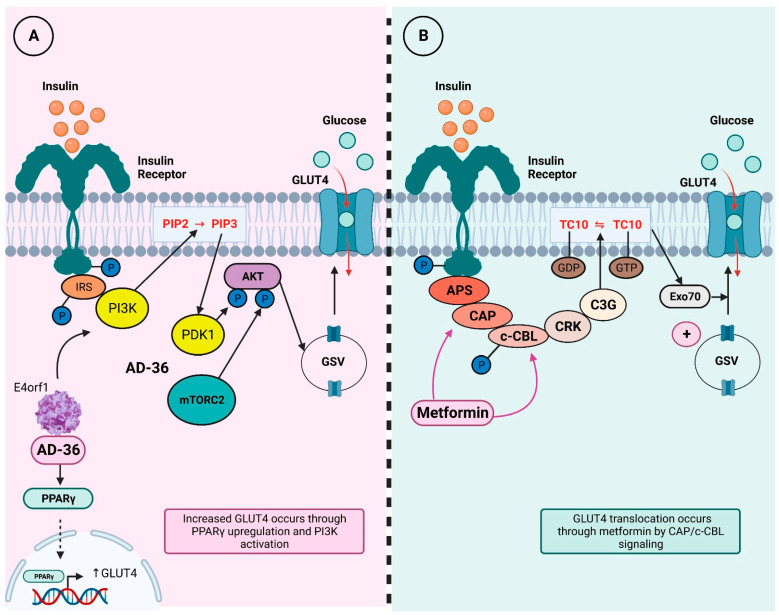
GLUT4 translocation through AD-36 and metformin. Insulin binding to its receptor activates a signaling pathway first regulated by a series of phosphorylation and then by adaptor protein recruitment; this pathway ultimately ends in GLUT4 translocation to cell membrane, which promotes glucose internalization. Abbreviations: IRS = insulin receptor substrate; PI3K = phosphoinositide 3-kinase; PIP2 = phosphatidylinositol-4,5-bisphosphate; PIP3 = phosphatidylinositol-3,4,5-trisphosphate; PDK1 = phosphoinositide-dependent kinase 1; Akt = protein kinase B; mTORC2 = mammalian target of rapamycin 2; AD-36 = adenovirus-36; e4orf1 = E4 open reading frame 1; PPAR-g = peroxisome proliferator-activated receptor-γ; GSV = GLUT4 storage vesicles; GLUT4 = glucotransporter 4; APS = adapter protein containing PH and SH2 domains; CAP = CBL-associated protein; c-CBL = cellular homolog of the transforming v-Cbl oncogene; CRK = adaptor protein CRK; C3G = guanine exchange factor C3G; TC10 = small GTP-binding protein TC10; Exo70 = subunit 70 of exocist complex. Modified from Sayem, 2018 [22]. (**A**). *In vitro* models have shown AD-36 interacts with insulin pathway and induces GLUT4 translocation through PPARγ upregulation, which favors GLUT4 transcription. Moreover, AD-36 E4orf1 gene also activates PI3K signaling and leads to GLUT4 translocation to cell membrane. (**B**). On the other hand, metformin intervenes in insulin signaling pathway in a different mechanism; it regulates CAP and c-CBL proteins and increases GLUT4 translocation from GSV to membrane.

Additionally, it has been observed that the E4 open reading frame 1 (E4orf1) gene of HAdV-36 upregulates the AKT/GLUT4 pathway, enhancing GLUT4 translocation, even in the presence of proinflammatory cytokines, allowing better glucose uptake in adipose tissue and skeletal muscle [20]. Increased GLUT4 translocation could enhance the effects of metformin, which increases AMPK-mediated GLUT4 translocation through the Cbl/CAP pathway, one of the two pathways that allow translocation of this glucose transporter [21,22]. Figure 1 illustrates the insulin signaling cascade and the effects of metformin and HAdV-36.

In light of the above, the present study aimed to investigate whether HAdV-36 influences the response to metformin monotherapy in obese patients with DM2. This work is pioneering in investigating whether virus-induced metabolic changes can be used to predict drug response.

## 2. Materials and Methods

### 2.1. Study Design

This study was a prospective cohort design. The follow-up period for each subject was 45 days, taking into account that metformin monotherapy can improve the glycemic profile within six weeks [23]. Two measurements were taken during the study period, one at baseline and one at the end of the estimated follow-up period.

### 2.2. Sample

A total of 103 volunteers were enrolled in this study, of which 36 (34.9%) were male and 67 (65.04%) were female, with a mean age of 48.82 ± 9.46 years old. All patients were referred from the Unidad Metabólica de Atención Nutricional Especializada (UMANE) in Ciudad Guzmán, Jalisco, Mexico. Only volunteers who met the following characteristics were included in this study: (a) subjects > 18 years of age with obesity (BMI ≥ 30 kg/m^2^) and new-onset DM2 who met American Diabetes Association (ADA) diagnostic criteria [24]; (b) patients with a recent medical indication for metformin as monotherapy at doses between 500 and 2550 mg/day; and (c) who started a nutritional plan with an energy deficit of 500–750 kcal/day for DM2 control.

Nutritional follow-up was provided by UMANE staff, with regular dietary check-ups every 15 days. Patients who did not attend all appointments were excluded from the study. The study was conducted between 1 November 2019 and 31 July 2020.

### 2.3. Biochemical Analyses

Volunteers’ blood samples were collected with at least eight hours of fasting for the determination of HAdV-36 serology and measurements of glycemic and lipid profiles. Blood was obtained by venipuncture of the forearm, using a vacuum-extraction blood sampling system. Samples for HAdV-36 serology were collected in 5 mL tubes without additives; immediately after collection, they were centrifuged at 3000 RPM for 10 min. The serum used for HAdV-36 identification was frozen at −20 °C until analysis. Virus identification was performed by enzyme-linked immunosorbent assay (ELISA) using the HAdV-36 ELISA antibody (AdV36-Ab, MyBioSource^®^ San Diego, CA, USA. Kit No. MBS705802) and a BiotekSynergy TH^®^ multimodal plate reader Winooski, VT, USA. The microplate reader was calibrated at a wavelength of 450 nm with which the optical density of each well was determined. Positive and negative values were calibrated according to the supplier’s instructions. According to the manufacturer’s instructions, the samples with optic density relation ≥2.1 compared with negative control were considered as positive samples for HAdV-36. The intra-assay precision mentioned by the supplier was within-test and between-test (inter-test precision), with a coefficient of variability of <15%.

Glucose and lipid analysis was performed on a semi-automated clinical chemistry analyzer Spinlab^®^ (manufacturer Spinreact^®^ Sant Esteve de Bas, Spain) The glucose hexokinase (ref: 1001201), cholesterol CHOD-POD Sant Esteve de Bas, Spain (ref: 41021) and triglyceride GPO-POD Sant Esteve de Bas, Spain (ref: 1001313) kits from Spinreact^®^ were used. All studies were performed at the Clinical Analysis Laboratory of the University Center of the South, University of Guadalajara.

### 2.4. Anthropometric Assessment

An anthropometric assessment was performed at baseline and the end of the study, employing bioelectric impedance analysis (BIA) using a Tanita Ironman BC-558 body composition monitor. Height was measured using a Seca 206 wall stadiometer, and waist circumference was measured using a Lufkin W606PMMX metal tape measure. Body mass index (BMI) was calculated as weight (kg)/height (m) squared to include obese subjects.

### 2.5. Bioethical Considerations

The present study was conducted in accordance with the “Ethical Principles for Medical Research Involving Human Subjects” of the Declaration of Helsinki and was approved by the local ethics committee of the Centro Universitario del Sur of the University of Guadalajara. All subjects signed a written informed consent prior to enrolment, in accordance with ethical guidelines.

### 2.6. Statistical Analysis

Data were presented as mean ± standard deviation (SD) or 95% confidence interval (95% CI) as appropriate. Normality analysis was performed using the Kolmogorov–Smirnov test. The comparison of quantitative variables between patients with the presence or absence of anti-HAdV-36 antibodies was performed using the *t*-test for parametric variables or the Mann–Whitney U test for nonparametric variables. Comparison of quantitative variables at baseline and the end of the cohort was performed using paired *t*-test or the Wilcoxon rank sum test, depending on the nature of the variables. A value of *p* < 0.05 was considered statistically significant. All analyses were performed using R version 4.1.2.

## 3. Results

Volunteers were first classified according to the presence or absence of anti-HAdV-36 antibodies, which revealed that 41.7% (*n* = 43) of the patients had anti-HAdV-36 antibodies (+HAdV-36). Once the groups were established (+HAdV-36 and −HAdV-36), biochemical and anthropometric variables were compared between them, both at baseline and after 45 days of treatment with metformin as monotherapy.

At baseline, the variables assessed were similar between groups, with the exception of glucose, which was significantly lower in the +HAdV-36 group. In terms of age, the anti-HAdV-positive group was, on average, about 4 years younger than the anti-HAdV-negative group.

In both groups, weight, BMI, body fat, serum lipids and waist/hip circumference decreased after metformin administration. The results are shown in Table 1.

Data were initially analyzed with the Kolmogorov–Smirnov (KS) test and subsequently evaluated with the paired *t*-test or the Wilcoxon rank sum test.

In addition, a statistical analysis was carried out, comparing the before and after metformin administration values of each variable according to the sex of the participants, both within and between groups.

In both groups, after 45 days of metformin treatment, there was a decrease in the study variables in both men and women. In particular, the HAdV-36-negative group of men had greater weight loss than the positive group and, as a result, weight-dependent variables such as BMI, waist and hip circumference were lower in the negative group.

In addition, the HAdV-36-negative group showed a greater decrease in blood glucose, but that is because the positive group started with an average blood glucose of 114 mg/dL and achieved normoglycemia with a slight decrease in blood glucose. The negative group started with a glucose level of 155.47 mg/dL and, although they lost 44.6 mg/dL on average, did not reach normoglycemia. The results can be seen in Table 2.

Data were initially analyzed with the Kolmogorov–Smirnov (KS) test and subsequently evaluated with the paired *t*-test or the Wilcoxon rank sum test.

## 4. Discussion

This is one of the few examples of research that studies the relationship between an infection and the response to a drug. The interaction between drug and infection has several causes. In some cases, drugs are metabolized by microorganisms before absorption; this phenomenon has been described in the gut microbiota, which, in some cases, carries out phase I and II reactions of drug metabolism [25].

It has also been described that some viruses, such as the Severe Acute Respiratory Syndrome Coronavirus (SARS-CoV-2), due to the inflammatory process it produces and, consequently, to the increase in cytokines, may decrease some cytochrome P450 (CYP) enzymes, such as CYP2B6 and CYP3A4, which may affect the pharmacokinetics of drugs metabolized by these enzymes [26].

The effect of HAdV-36 on the response to metformin was investigated in this study. Although the virus has not been shown to alter the metabolism of the drug, the interaction may exist, as HAdV-36 causes metabolic changes similar to those of TZD and affects the metabolic pathways in which metformin acts and there may be a synergistic effect between the virus and the drug [13].

In terms of seroprevalence, antibodies against HAdV-36 were found in 41.7% of the population studied, which is lower than the 73.9% reported in a Mexican obese children population [27]. However, it is higher than that reported in European populations, where seropositivity of 26.4% was reported in Polish HIV-infected patients; 26.5% in Czech adolescents; and <20% in Swedish patients [28,29,30].

Regarding the gender distribution of seropositivity for HAdV-36 antibodies, the prevalence of antibodies was found to be higher in women than in men (60.5% vs. 39%, respectively), similar to data reported in the United Arab Emirates (53% in women and 41% in men) [31]. Similarly, in Italian patients with nonalcoholic fatty liver disease (NAFLD), seropositivity was 56% in women and 44% in men [32]. In the present study, these differences may be due to the fact that more women than men agreed to take part in the study. This was probably also the case in the studies cited above. On the other hand, the higher seroprevalence of HAdV-36 antibodies in women may be explained by natural differences in the functioning of the immune system, because women have a stronger humoral response and produce higher and longer-lasting antibody titers to infection or vaccination, including some SARS-CoV-2 vaccines [33]. Considering that HAdV-36 is detected through its antibodies, gender could be a determining factor.

The characteristics of the study groups (+HAdV-36 and −HAdV-36) were as expected according to the selection criteria. The groups were very homogeneous and no significant differences were found for most variables. BMI, body fat percentage, and waist and hip circumferences were very similar between the study groups. Plasma triglyceride levels were higher in the +HAdV-36 group, but the difference was not significant. Cholesterol levels were lower in the seropositive group but not significantly so.

Despite these similarities between groups, glucose was lower in the +HAdV-36 group (120.5 ± 50.06 vs. 148.3 ± 69.83). This finding is consistent with *in vivo* and *in vitro* studies that have identified cell signaling pathways modulated by HAdV-36 to influence cellular glucose metabolism and improve glycemic control [10,34].

Previously, HAdV-36 seropositivity was associated with lower fasting glucose and insulin levels only in normal-weight men and women [35], which differs from our results, but this difference can be explained because, in our study population, only patients with newly diagnosed DM2 and obesity were included, making our population more homogeneous for comparison purposes.

After treatment with metformin and a caloric-deficit diet, significant changes were observed. Both groups lost weight, with the +HAdV-36 group losing an average of 3.2% of body weight, while the −HAdV-36 group lost 2.7%; although the seropositive group lost more weight on average, the difference was not statistically significant. The effect of metformin on weight has been reported in several publications [36,37,38]. In a study that looked at the effect of metformin in combination with a diet plan, the majority of people with diabetes and prediabetes lost an average of 6.5% of their body weight over six months [38].

Considering HAdV-36 as a benchmark, there are no other studies reporting weight loss in a population similar to this study; however, it has been reported that people lose weight similarly after multidisciplinary weight loss interventions regardless of HAdV-36 serotype [39,40].

In the present study, we observed that the HAdV-36-seropositive group of men lost less weight on average than the seronegative men; this difference was not observed in women. Nevertheless, the difference in weight loss observed in men may possibly be due to physical activity. In the trial, there was good follow-up of the diet plan, adherence to the diet, and medication use and the age of the groups was similar. However, no specific exercise plan was provided as part of the treatment, nor was a tool used to assess physical activity levels. It is, therefore, possible that this variable was a factor in the differences in outcomes.

Consistent with weight loss, decreases in BMI, body fat percentage and waist and hip circumference were observed in all study groups. In terms of BMI and waist and hip circumference, it was observed that the HAdV-36-negative group performed better. This is because the HAdV-36-positive men lost less weight. In the women’s group, it was also observed that the range of hip and waist measurements was very wide. This is mainly because the age range of the women included (40–55 years) includes both postmenopausal and premenopausal women, who have a different distribution of body fat due to hormonal changes [41].

HAdV-36-positive subjects had lower glucose levels at baseline. However, this result was expected as the virus increases glucose uptake by skeletal muscle cells [42].

After metformin administration, we found that all groups had significantly lower glucose levels, which is in line with the findings previously reported regarding the fasting glucose levels and insulin resistance decreased after six weeks of metformin therapy, [23]. However, we expected the decrease in glucose to be greater in the HAdV-36-positive group, but this was not the case; even in the sex-adjusted analysis we found that the HAdV-36-negative group of men decreased glucose levels even more than the positive group.

At baseline, there was no difference in cholesterol or triglyceride levels across study groups. In most of the study patients, cholesterol was normal and triglycerides were slightly higher. The reduction in cholesterol and triglyceride levels following metformin treatment was considerable in both groups, as has been described previously [43,44].

It is recommended that future studies test glucose values on multiple occasions to further assess the progression of the decline in glucose levels. It is also recommended to perform A1C and oral glucose tolerance tests in addition to fasting glucose, in order to have a more accurate assessment [45]. On the other hand, due to the limits imposed by the COVID-19 pandemic, further follow-up and laboratory testing were not possible in this study.

It is important to consider that some of the study results may be influenced by the coincidence between the study period and the COVID-19 pandemic. During this period, there were important changes in lifestyle, both in terms of dietary habits and physical activity [46,47]. As these factors may affect or determine the variables analyzed in the study, they must be taken into account for future studies.

Finally, ELISA was used in this study to identify antibodies against HAdV-36. Serological tests are by far the most widely used in human HAdV-36 research, and ELISA is one of the most sensitive tests but not the most specific [48]. Because there are reports that several adenoviruses have been associated with obesity [49], in future research, it is advisable to complement the ELISA with tests with higher specificity for HAdV-36, such as serum neutralization assay (SNA) or polymerase chain reaction (PCR) from fecal samples or adipose tissue, depending on availability, characteristics of the study population and research objectives [48]. The use of combined tests can help to improve the strength of the evidence that will be provided by future research.

## Figures and Tables

**Table 1:** Comparison of biochemical and anthropometric variables between +HAdV-36 and −HAdV-36 groups.

Variable	Measuring	+HAdV-36 (*n* = 43)	−HAdV-36 (*n* = 60)	*p*-Value
Weight (Kg)	Baseline	95.4 ± 16.6	92.2 ± 15.9	0.151
	After 45 days	92.5 ± 16.3	89.8 ± 15.3	0.146
	Difference	−2.9 ± 1.7	−2.4 ± 1.5	0.791
IMC (kg/m^2^)	Baseline	35.5 ± 4.4	34.5 ± 5.5	0.573
	After 45 days	33.5 ± 5.3	33.5 ± 4.2	0.555
	Difference	−2 ± 0.7	−1 ± 0.6	0.763
Body fat (%)	Baseline	35.6 ± 7.4	37.3 ± 5.9	0.212
	After 45 days	32.3 ± 6.7	34.1 ± 5.7	0.170
	Difference	−3.3 ± 2.3	−3.2 ± 1.3	0.873
Waist circumference (cm)	Baseline	105.4 ± 14.2	103.5 ± 12.5	0.393
	After 45 days	102.7 ± 13.9	100.51 ± 12	0.373
	Difference	−2.7 ± 1.5	−3.02 ± 1.56	0.31
Hip circumference (cm)	Baseline	106.04 ± 17.8	103.9 ± 15.6	0.542
	After 45 days	99.9 ± 25.1	102.3 ± 14.8	0.964
	Difference	−6.2 ± 22.7	−1.7 ± 44.64	0.126
Glucose (mg/dL)	Baseline	120.5 ± 50.06	148.3 ± 69.8	0.005 *
	After 45 days	97.7 ± 25.3	107.09 ± 33.3	0.029 *
	Difference	−22.8 ± 26.7	−41.2 ± 44.6	0.006 *
Total cholesterol (mg/dL)	Baseline	168.7 ± 42.4	181.8 ± 59.1	0.644
	After 45 days	139.8 ± 27.7	155.9 ± 42.8	0.055
	Difference	−28.9 ± 30.08	−25.8 ± 36.4	0.404
Triglycerides (mg/dL)	Baseline	197.3 ± 104.3	185.7 ± 110.6	0.387
	After 45 days	160.6 ± 60.8	150.9 ± 57.6	0.350
	Difference	−36.7 ± 59.5	−34.8 ± 63.8	0.866
Age (years)		46.7 ± 9.8	50.95 ± 9.12	0.24 *

* Statistically significant.

**Table 2:** Comparison within and between groups of patients according to sex, before and after metformin administration.

		−HAdV-36 *n* = 60				+HAdV-36 *n*= 43			
Variables	Measuring	Men *n* = 19		Women *n* = 41		Men *n* = 17		Women *n* = 26	
		Mean	*p*-Value	Mean	*p*-Value	Mean	*p*-Value	Mean	*p*-Value
Age (years)	-	51.9 ± 7.3	-	50.5 ± 10.4	-	46.35 ± 11.4	-	46.8 ± 9.39	-
Weight (Kg)	Baseline	100.5 ± 13.6	0.001 *	86.2 ± 11.1	0.001 *	105.6 ± 15.4	0.001 *	89.6 ± 14	0.001 *
	After 45 days	97.5 ± 13.2		84.1 ± 10.6		103.5 ± 15.7		87 ± 13.2	
	Differences in men	−2.9 ± −0.3				−1.9 ± 0.3			0.039 *
	Differences in women			−2.1 ± −0.9				−2.6 ± −0.8	0.25
IMC (kg/m^2^)	Baseline	34.07 ± 7.07	0.001 *	35.5 ± 4.6	0.001 *	34.6 ± 4.5	0.001 *	35.5 ± 4.4	0.001 *
	After 45 days	33.07 ± 6.94		34.6 ± 4.3		33.9 ± 4.7		34.4 ± 4.1	
	Differences in men	−0.9 ± −0.13				−0.6 ± −0.2			0.028 *
	Differences in women			−0.8 ± −0.29				−1.1 ± −0.3	0.395
Body fat (%)	Baseline	33.4 ± 4.6	0.001 *	38.5 ± 5.1	0.001 *	31.0 ± 4.9	0.001 *	38.5 ± 7.4	0.001 *
	After 45 days	30.6 ± 4.58		36 ± 4.8		28.7 ± 4.4		35.6 ± 6.8	
	Differences in men	−2.7 ± −0.02				−2.5 ± −0.5			0.326
	Differences in women			−2.5 ± −0.3				−2.8 ± −0.6	0.456
Waist circumference (cm)	Baseline	105.6 ± 14.7	0.001 *	102.2 ± 10.6	0.001 *	103.5 ± 13.1	0.001 *	105.9 ± 14.8	0.001 *
	After 45 days	101.4 ± 14.4		99.5 ± 10.1		101.4 ± 13.2		102.7 ± 14.4	
	Differences in men	−4.2 ± −0.3				−2.1 ± 0.1			0.003 *
	Differences in women			−2.7 ± −035				−3.1 ± −0.4	0.211
Hip circumference (cm)	Baseline	96.2 ± 12.5	0.001 *	106.8 ± 15.8	0.001 *	97.1 ± 18.9	0.434	110.8 ± 14.4	0.081
	After 45 days	94.8 ± 12.03		104.8 ± 14.8		96.8 ± 18.8		100.6 ± 28.4	
	Differences in men	−1.3 ± −0.47				−0.2 ± −0.1			0.035 *
	Differences in women			−2 ± 1				−10.2 ± 14	0.073
Glucose (mg/dL)	Baseline	155.47 ± 63.5	0.006 *	139.7 ± 55.7	0.001 *	114 ± 63.8	0.136	121.5 ± 36.6	0.001 *
	After 45 days	110.8 ± 32.2		102.6 ± 26.3		95.8 ± 19		98 ± 29	
	Differences in men	−44.6 ± −31.3				−18.1 ± −44.8			0.016 *
	Differences in women			−37.1 ± −29.4				−23.5 ± −7.6	0.186
Total cholesterol (mg/dL)	Baseline	169.3 ± 42.6	0.002 *	180.6 ± 50.6	0.001 *	158 ± 52.8	0.023 *	173.3 ± 32.2	0.001 *
	After 45 days	152.6 ± 34.9		154.5 ± 38.7		136.2 ± 28.8		140.5 ± 26.6	
	Differences in men	−16.6 ± 7.7				−21.7 ± −24			0.661
	Differences in women			−26.1 ± −11.9				−32.7 ± −5.6	0.409
Triglycerides (mg/dL)	Baseline	210.86 ± 126.2	0.007 *	164.5 ± 71.6	0.003 *	206.7 ± 120.8	0.012 *	176.9 ± 58.3	0.004 *
	After 45 days	161.7 ± 62.5		141.4 ± 38.8		153.4 ± 56		155.3 ± 39.5	
	Differences in men	−49.16 ± −63.7				−53.2 ± −64.8			0.867
	Differences in women			−23.1 ± −32.8				−21.6 ± −18.8	0.891

* Statistically significant.

## Data Availability

The data presented in this study are available upon request from the corresponding author. The data are not publicly available due to the confidentiality agreement with the study subjects.
